# CD147 monoclonal antibody mediated by chitosan nanoparticles loaded with α-hederin enhances antineoplastic activity and cellular uptake in liver cancer cells

**DOI:** 10.1038/srep17904

**Published:** 2015-12-07

**Authors:** Rong Zhu, Chun-ge Zhang, Yang Liu, Zhi-qiang Yuan, Wei-liang Chen, Shu-di Yang, Ji-zhao Li, Wen-jing Zhu, Xiao-feng Zhou, Ben-gang You, Xue-nong Zhang

**Affiliations:** 1 Department of Pharmaceutics, College of Pharmaceutical Sciences, Soochow University, Suzhou 215123, People’s Republic of China; 2 College of Radiological Medicine and Protection, Soochow University, Suzhou 215123, People’s Republic of China; 3 Changshu Hospital of Traditional Chinese Medicine, Changshu 215500, People’s Republic of China

## Abstract

An antibody that specifically interacts with an antigen could be applied to an active targeting delivery system. In this study, CD147 antibody was coupled with α-hed chitosan nanoparticles (α-Hed-CS-NPs). α-Hed-CS-CD147-NPs were round and spherical in shape, with an average particle size of 148.23 ± 1.75 nm. The half-maximum inhibiting concentration (IC50) of α-Hed-CS-CD147-NPs in human liver cancer cell lines HepG2 and SMMC-7721 was lower than that of free α-Hed and α-Hed-CS-NPs. α-Hed-induced cell death was mainly triggered by apoptosis. The increase in intracellular accumulation of α-Hed-CS-CD147-NPs was also related to CD147-mediated internalization through the Caveolae-dependent pathway and lysosomal escape. The higher targeting antitumor efficacy of α-Hed-CS-CD147-NPs than that α-Hed-CS-NPs was attributed to its stronger fluorescence intensity in the tumor site in nude mice.

Currently, clinical therapies for liver cancer focus on operation and radiotherapy. A systematic standard treatment regimen has not been established because of limited chemotherapy drug varieties and significant side effects. In this regard, targeted cancer therapy has gained increasing attention because of its high specificity, significant efficacy, and minimal adverse reactions. Among the top 10 best-selling drugs in 2013, five types of monoclonal antibodies contain adalimumab for arthritis^1^, infliximab and rituximab for non-Hodgkin lymphoma^2^, bevacizumab for metastatic colorectal cancer, and herceptin for metastatic breast cancer^3^,^4^. Furthermore, adalimumab, infliximab, and rituximab have been the top three best-selling drugs over the recent years, indicating that monoclonal antibodies exhibit great advantages in tumor therapy.

α-hederin (α-hed) was extracted and purified from total saponins of *Pulsatilla chinensis* (Bge.) Regel^5^ Rooney^6^,^7^ found that α-hed exhibited significant cytotoxicity and induced apoptosis of cancer cells, such as colon cancer cell line HT-29, pancreatic cancer cell line Paca-1, and lung cancer cell line A549. Another study reported that α-hed affected growth inhibition and pro-apoptosis in breast cancer cells^8^. This paper is the first to report that α-hed could depolarize the mitochondrial membrane potential, resulting in release of Apaf-1 and cytochrome C from the inter-membrane space to the cytosol. As α-hed can also cause strong contraction to smooth muscles^9^, it may be involved in calcium activation. Given that α-hed is lipophilic and presents low bioavailability and poor oral absorption, this work focused on improving its efficacy by entrapping it into nanoparticles (NPs).

Chitosan (CS), one kind of hydrogel-forming polymers^10^, can be widely obtained and used to entrap lipophilic and hydrophilic compounds because of its good biocompatibility, biodegradability, nontoxicity, film formation, permeability, non-allergic, and plasticity^11^,^12^. The pharmaceutical application of CS and chemical analogs was quite extensive, such as topical delivery, ocular delivery and coating material^13^. As a permeable polymer, CS can form interpenetrating polymer network with guar gum^14^,^15^. Silymarin, a hepatoprotective drug^16^, could be entrapped into CS through ionic gelation for passive targeting delivery. As a biodegradable material, CS can encapsulate antigen or DNA to protect them from damage or form complexes with DNA for gene delivery^17^,^18^,^19^,^20^. As a siRNA delivery nanocarrier, the transfection efficiency could be as high as 89%^21^.

Deacetylated CS contains active hydroxyl and amino groups and exhibits numerous chemical reactions, such as PEGylation, hydroxyethylation, carboxymethylation, and cyanoethylation. The deacetylation, molecular weight and chemical modification of CS affected transfection efficiency of siRNA^22^. Its modified analogs have been widely used for insulin therapy^23^. In the presence of the asialoglycoprotein receptor, lactose and galactose could be modified to CS, which functions as ligands for positive targeting delivery of drugs or genes^24^,^25^,^26^,^27^,^28^,^29^. Folate-conjugated CS could also be utilized as a part of vector to enhance tumor targeting^30^. Moreover, methylation to CS could increase the potential of NPs to easily approach the tumor^31^.

As most recent studies have focused on administering antibodies entrapped into vectors as drugs, few works have combined antibodies with lipophilic drug-loaded NPs. In previous research, α-Hed-CS-NPs were prepared through emulsion solvent diffusion^32^ and NPs with appropriate particle size can be passively delivered to specific target organs, tissues, and cells. In the present work, α-Hed-CS-NPs were modified with CD147 antibody to obtain positive targeting and enhance antitumor activity. CD147 antibody was overexpressed in liver cancer cells, such as SMMC-7721^33^. CD147 antibody could also be used as a drug for HCC treatment because it regulates the expression levels of MMP2 and CD31 or induces tumor necrosis^34^. Nevertheless, few studies reported the active targeting of CD147 antibody mediated by antigens. In this paper, α-Hed-CS-NPs and α-Hed-CS-CD147-NPs were systematically and integrally compared. This work aimed to formulate a suitable targeting delivery system for α-Hed in liver cancer and determine its anti-proliferative ability and targeting efficacy (Fig. 1). *In vitro* antitumor activity, endocytosis mechanism, competitive inhibition, cellular uptake, subcellular localization, and *in vivo* tumor targeting were further investigated.

## Methods

### Materials and cell lines

α-Hederin was extracted and purified in our laboratory to obtain a drug purity of 90% as detected by HPLC. CS with a molecular weight (MW) of around 8 kDa-10 kDa and deacetylation degree of 90.9% was purchased from Xingcheng Biochemical Co, Ltd. (Nantong, China). Galactosylated CS (GC) was synthesized based on a published method^25^. CD147 was obtained from BD (BD Pharmingen), and HRP-conjugated goat anti-mouse IgG antibody was purchased from Abgent (Flanders Court, San Diego). 1-Ethyl-3-(dimethylaminopropyl) carbodiimide hydrochloride (EDC), N-hydroxysuccinamide (NHS), and *o*-phenylenediamine were purchased from Sinopharm Chemical Reagent Co., Ltd. (Shanghai, China). Fluorescein isothiocyanate (FITC) and 3-(4, 5-Dimethylthiazol-2-yl)-2, and 5-diphenyltetrazolium bromide (MTT) were purchased from Sigma–Aldrich (St. Louis, Missouri). Annexin V–FITC and propidium iodide (PI) were obtained from Beyotime (Shanghai, China).

The human hepatoma cell lines HepG2 and SMMC-7721 and the hepatic stellate cell line (HSC) were acquired from the Teaching and Research Section of Pharmacology, Soochow University. HepG2 and SMMC-7721 were cultured in RPIM-1640 medium supplied with 10% FBS (v/v). HSC was grown in DMEM medium supplemented with 10% NBS (v/v), penicillin (100 U/mL), and streptomycin (100 U/mL). The cells were maintained in a humid incubator (5% CO_2_) at 37 °C.

Male nude mice (body weight, 18–22 g) were purchased from the Experimental Animal Center of Soochow University (Suzhou, China). All nude mice were grown according to the criteria of the National Institutes of Health for the care and use of laboratory animals. All animal experiments were conducted according to the protocols approved by the Institutional Animal Care.

### Preparation of α-Hed-CS-CD147-NPs

Modification was conducted by activating the carboxyl group of the antibody through EDC–NHS method^35^. The conjugation was also reported occurring similarly in RGD and LDL^36^. Briefly, 30 μL of CD147 (0.5 mg/mL CD147 in 0.1 M PBS, pH 7.4) was added to 5 mL of PBS (0.1 M, pH 5.8). The suspension was then added with 30 mg of EDC and 15 mg of NHS. After 30 min, the suspension was added dropwise with 10 mg of α-Hed-CS-NPs^37^ dissolved in 3 mL of PBS (0.1 M, pH 5.8) at room temperature (25 ± 5 °C) under sustained magnetic stirring for 12 h. The solution was ultra-centrifuged using the Optima MAX centrifuge (Beckman Coulter Inc., Fullerton, California) at 54000 rpm and 4 °C for 45 min to remove redundant EDC and unmodified CD147. The solution was centrifuged twice using the previously described conditions, and modified NPs were obtained as precipitates. The supernatant was collected while washing the product to determine the amount of residual CD147 by using a BCA protein assay kit (Thermo Scientific, Pierce). For cell experiments, FITC-labeled CS NPs were also modified with CD147 through the same method.

### Physico-chemical characterization of NPs

The particle size and zeta potential of NPs were measured using a particle size and zeta potential analyzer (NICOMP 380ZLS, Santa Barbara, USA). The morphological characteristics of NPs were investigated under a transmission electron microscope (TEM, JEM-1230, Tokyo, JEOL, Japan). A drop of the prepared NP colloid solution was placed onto a carbon film-coated round copper grid and then air dried. Finally, negative staining was used for TEM observation.

### Immunoactivity assay

The immunoactivity of CD147 monoclonal antibody was tested to determine its conserved activity in α-Hed-CS-CD147-NPs. CD147 antibody and α-Hed-CS-CD147-NPs were added to the antigen (SMMC-7721 cells) for ELISA assays. Briefly, 5 × 10^3^ SMMC-7721 cells per well were cultured into sterile 96-well ELISA plates for 24 h. The cells were washed thrice, air dried, fixed for 30 min at 4 °C with pre-cooled 4% paraformaldehyde solution, and sealed with 300 μL of 1% BSA per well at 37 °C. After sealing for 1 h, the samples were washed twice with PBST (containing 0.05% Tween-20 in PBS) to remove the residual medium. The samples were then incubated with 100 μL of gradient concentrations of CD147 or α-Hed-CS-CD147-NPs for 1 h at 37 °C and co-cultured with HRP-conjugated goat anti-mouse IgG antibody (dilution degree = 1:2000) for another 1 h at 37 °C. After washing twice, antigen-CD147-HRP compound was reacted with 100 μL of o-phenylenediamine for 20 min in the dark and then terminated using 50 μL of 2 M H_2_SO_4_ per well. Optical density (OD) was determined at 490 nm by using a microplate reader (Model ELx 800, Bio-Tek, Winooski, Vermont). Each concentration of CD147 was prepared in five parallel wells, and buffer-treated cells were set as control. The activity of CD147 was determined as the relative activity conservation rate based on the actual values measured from the given standard curve.

### Fourier transform infrared (FTIR) spectrum evaluation

The IR spectra of α-Hed-CS-NPs, CD147, and α-Hed-CS-CD147-NPs were obtained using an FTIR spectrometer (Varian, Palo Alto, California) to investigate the modification of CD147 antibody on the surface of α-Hed-CS-NPs. Briefly, the freeze-dried samples were blended with potassium bromide to obtain pellets for FTIR detection.

### *In vitro* cytotoxicity studies

The *in vitro* cytotoxicity of free α-Hed, α-Hed-CS-NPs, and α-Hed-CS-CD147-NPs were tested on HepG2, SMMC-7721, and HSC cells through MTT assay. Briefly, HepG2 cells were (8 × 10^4^ cells per well) seeded into 96-well plates and incubated for 24 h. The medium was replaced with sterile samples (free α-Hed, α-Hed-CS-NPs, and α-Hed-CS-CD147-NPs) at various predetermined concentrations, and the cells were incubated for another 24 h. Each well was added with 20 μL of 5 mg/mL MTT in PBS and then cultured for 4 h. Finally, 100 μL of DMSO was added instead of the above medium to dissolve the formazan crystals. OD was determined at 490 nm by using a microplate reader (Model ELx 800). Cell viability was evaluated as the percentage of drug-treated cells to medium-treated control cells.

### Apoptosis assays

Cell apoptosis was detected using an Annexin V-FITC apoptosis assay kit. Briefly, 5 × 10^5^ HepG2 cells were seeded in six-well plates and incubated for 24 h. The cells were treated with vacant CS-CD147-NPs, free α-Hed (50 μg/mL), α-Hed-CS-NPs (50 μg/mL), and α-Hed-CS-CD147-NPs (50 μg/mL) for 24 h. After incubation, the cells were washed twice with 5 mL of pre-cooled PBS, resuspended in binding buffer, and mixed with Annexin V and PI at room temperature for 15 min. Finally, cell apoptosis induced by drug treatment was directly analyzed with FACS (BD FACS Calibur).

### Cellular uptake by flow cytometry

The cellular uptake of NPs was also determined by FACS. Briefly, HepG2 cells in logarithmic growth phase were collected, seeded in six-well plates at 5 × 10^5^ per well, and then incubated with FITC-labeled α-Hed-CS-NPs and α-Hed-CS-CD147-NPs for 24 h. Afterward, the cells were washed twice with PBS and analyzed by FACS. In addition, HSC cells were incubated with FITC-labeled α-Hed-CS-NPs and α-Hed-CS-CD147-NPs for 24 h to confirm the influence of CD147-mediated active targeting on cellular uptake.

### Effects of competitive inhibition on cellular uptake

Cellular competitive inhibition uptake was determined through flow cytometry to confirm the effect of CD147-mediated active targeting. Briefly, 5 × 10^5^ HepG2 cells per well were seeded into six-well plates and incubated for 2 h with excess CD147 as competitive inhibitor. The effect of excess CD147 on HepG2 cell uptake was assessed as the relative uptake of NP-treated control cells. The feasibility of the tests was illustrated with the introduction of α-Hed-GC-NPs pretreated with excessive lactose as positive control cells. GC was synthesized in our previous study^25^.α-Hed-GC-NPs were also prepared through emulsion solvent diffusion method.

### Intracellular binding and localization of NPs

Cellular binding and uptake was observed under a confocal microscope. The sterilized coverslips were plated on the bottom of six-well plates and seeded with 5 × 10^4^ HepG2 cells per well. After reaching around 70% degree of cell fusion, FITC-labeled α-Hed-CS-NPs and α-Hed-CS-CD147-NPs (primary antibody) were added and incubated in the dark for 1, 2, and 4 h. The medium was removed, and the cells were rapidly washed twice with pre-cooled PBS. The cells were fixed with 4% neutral formaldehyde solution in PBS (0.1 M, pH 7.4) for 10 min at 4 °C and then washed twice. The washed cells were co-cultured with the second PE-labeled goat anti-mouse fluorescence antibody (secondary antibody) for 30 min to detect the existence of CD147 in α-Hed-CS-CD147-NPs. After washing twice, the cell nucleus was stained with 1 mL of Hoechst33258 (10 μg/mL) in PBS (0.1 M, pH 7.4) for 10 min. Finally, the coverslips seeded with HepG2 cells were inverted to slides for observation under a confocal microscope.

### Effects of endocytosis inhibitors on cellular uptake

The effects of endocytosis inhibitors on cellular uptake were evaluated with FACS. Briefly, 5 × 10^5^ HepG2 cells per well were plated onto six-well plates pretreated with sodium azide^38^ (NaN_3_, 200 μg/mL), sucrose^39^ (0.45 M), chlorpromazine^40^ (7 μg/mL), methyl-β-cyclodextrin^41^ (MβCD, 3 nM), and wortmannin (500 nM) for 1 h. After rapid washing twice, the cells were incubated with FITC-labeled NPs for 2 h. The samples were resuspended for FACS analysis. The effects of endocytosis inhibitors were evaluated as the relative cell uptake rate with non-treated cells as background and NP-treated cells as positive control. NP-treated cells were regarded as 100% cellular uptake.

### Intracellular colocalization of NPs

The location of NPs in specific organelles was observed under a confocal microscope to determine cellular distribution^42^. Briefly, 5 × 10^4^ HepG2 cells were plated to sterile coverslips and incubated with α-Hed-CS-CD147-NPs for 2 h. The lysosome, Golgi bodies, and endoplasmic reticulum were stained with lyso-tracker red, Golgi-tracker red, and ER-tracker red, respectively. The cells were then co-cultured with 1 mL of Hoechst33258 (10 μg/mL) in PBS (0.1M, pH 7.4) for 10 min, and the coverslips were placed upside down onto slides for investigation under a confocal microscope.

### *In vivo* NIR fluorescence real-time imaging of nude mice containing HepG2

HepG2 cells were cultured twice at 37 °C with 5% CO_2_. Cell concentration was adjusted to 5 × 10^8^ per milliliter, and 0.2 mL of the cell suspension medium was injected to the right axillary subcutaneous of each mouse. Cy7 and the drug in organic phase were simultaneously injected into aqueous phase to prepare Cy7-labeled NPs. After the tumor volume increased to a certain degree, 5 mg/kg of α-Hed entrapped into Cy7-labeled α-Hed-CS-CD147-NPs was administered to nude mice via tail vein. Fluorescence distribution was recorded at predetermined time points by using an *in vivo* imaging system (IVIS Lumina II; Caliper Life Sciences, Hopkinton, MA, USA).

## Statistical analysis

All values were presented as mean ± standard deviation (SD). Values were analyzed by one-way ANOVA and post-hoc Tukey’s test for paired comparison (SPSS 16; SPSS Chicago, Illinois). Statistical significance was set at *p < 0.05 and **p < 0.01 or ^#^p < 0.05 and ^##^p < 0.01.

## Results and Discussion

### Characterizations of α-Hed-CS-CD147-NPs

**Figure 2:**
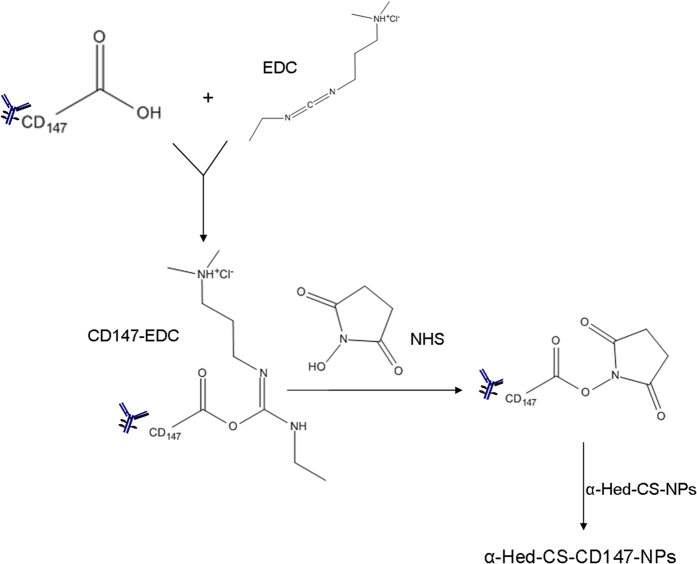
Synthesis of α-Hed-CS-CD147-NPs.

Figure 2 illustrates the interpretative exhibition of α-Hed-CS-NP surface modified with CD147. α-Hed-CS-NPs were prepared through emulsion solvent diffusion with EDC and NHS as conventional catalysts of amide reaction. During the reaction, *o*-acylisourea ester intermediate was generated after the carboxyl groups of CD147 antibody were activated by EDC. NHS was also added to produce semi-stable amine-reactive NHS-ester because of the instability of the intermediate in aqueous solution. At pH 5.8, α-Hed-CS-NPs containing the activated amide group were added dropwise to generate stable amide bonds and prepare α-Hed-CS-CD147-NPs. The encapsulation efficiency and the release property of α-Hed-CS-CD147-NPs (data not shown) was similar to α-Hed-CS-NPs. Briefly, the encapsulation efficiency were 75.63 ± 4.06%, besides, in pH 6.8 and below, α-Hed was released lower than 60% in 10 h. However, it was released more than 80% under pH 7.4 after 10 hours.

Standard curve equation was constructed through curve fitting using the concentration of CD147 antibody (X-axis) and the corresponding absorption value at 490 nm (Y-axis) to determine antibody activity. Four-parameter curve fitting was then applied to determine the modification amount as *y* = [(*A*–*D*)/[1 + (*x*/*C*) ^*B*] + *D*] (*R*^2^ = 0.9972, *A* = 0.3366, *B* = 1.1781, *C* = 11.3371, and *D* = 1.2133); in this equation, the linear range was set at 0.5–20 g/mL. Activity assay demonstrated that the conserved activity after modification was 57.07% ± 0.60%. The relatively low conservation could be attributed to the participation of part of the carboxyl groups in the active functional domain in the amide reaction; for example, the carboxyl groups in the side chain of proline and aspartic acid.

**Figure 3:**
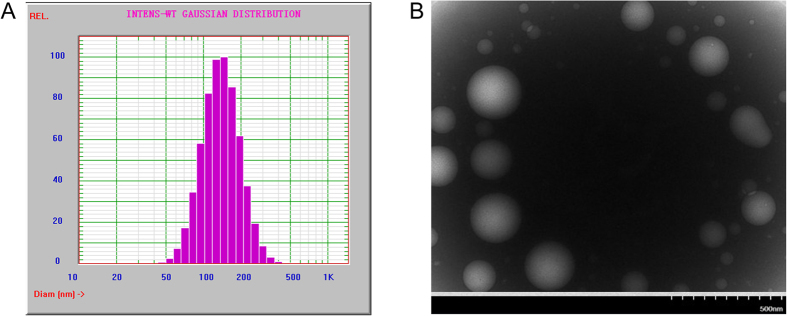
Characterization of particle size and morphology of α-Hed-CS-CD147-NPs. (**A**) Size distribution and (**B**) TEM micrograph.

The TEM graph of α-Hed-CS-CD147-NPs in Fig. 3B revealed that the prepared NPs were smooth and round, formed a solid sphere, and exhibited particle sizes ranging from 50 nm to 300 nm. Figure 3A demonstrated the particle size distribution of α-Hed-CS-CD147-NPs, with an average size of 148.23 ± 1.75 nm and PDI of 0.109 ± 0.010. As shown in the TEM graph, the particle size was larger than 200 nm, which could be attributed to the sample preparation process. During air drying, NPs shrunk and collapsed, resulting in loss of the hydration layer on the particle surface. In addition, the potential of NPs decreased from 20.74 ± 0.75 mV to 10.48 ± 0.79 mV. The modification of CD147 on the surface of α-Hed-CS-NPs covered the free amino groups, thus inducing nanoparticle aggregation and decreasing the potential. Low-power ultrasound was also adopted during resuspension of the centrifuged NPs to efficiently prevent aggregation. The maximum amount of CD147 antibody modified to NPs was 6.550 ± 0.234 μg/mg α-Hed-CS-NPs. The restriction in the degree of modification was attributed to the limited amount of free carboxyl in CD147, which could efficiently react with the amino groups, and the existence of steric hindrance caused by modification.

**Figure 4:**
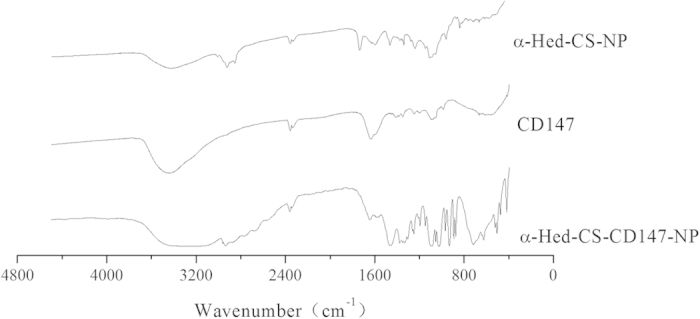
FTIR characterization of α-Hed-CS-CD147-NPs.

FTIR characterization and analysis were conducted to investigate the chemical substitution, elimination, or entrapped concealing of certain chemical groups. These processes occurred during the formation of NPs in the presence of band broadening or shifting caused by the modification of CD147 antibody on the surface of α-Hed-CS-NPs (Fig. 4). As shown in the figure, the characteristic absorption peak of α-Hed-CS-NPs at 1598 cm^−1^ (N-H vibration deformation in amine) shifted to a lower wavenumber (1570 cm^−1^) and became weaker in α-Hed-CS-CD147-NPs. A peak was also observed at 716 cm^−1^ (N-H out-of-plane vibration deformation in amide), indicating the modification of CD147 to NPs.

### *In vitro* cytotoxicity and apoptotic effects

**Figure 5:**
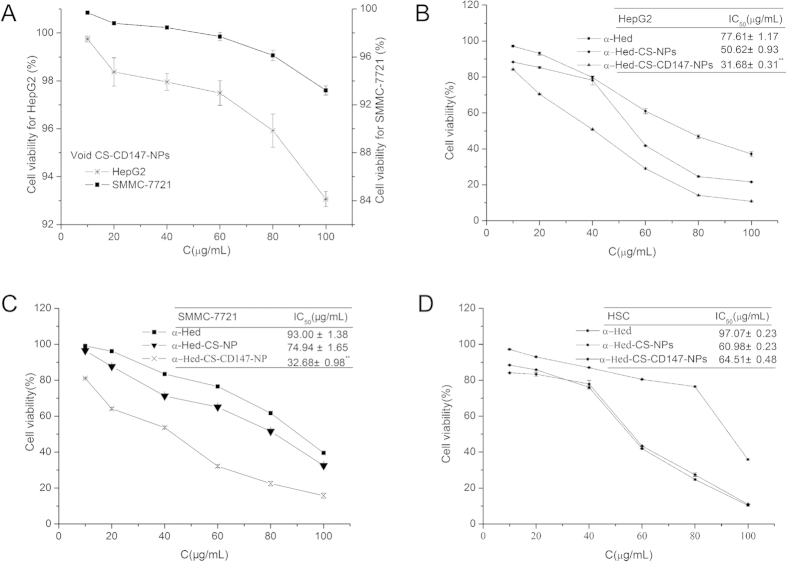
*In vitro* cytotoxicity of different formulations. (**A**) Cytotoxic effect of void CS-CD147-NPs on HepG2 and SMMC-7721 cells after incubation for 24 h (*n* = 3); (**B**) cytotoxic effect of α-Hed, α-Hed-CS-NPs, and α-Hed-CS-CD147-NPs on HepG2 cell line after incubation for 24 h (*n* = 3); (**C**) cytotoxic effect of α-Hed, α-Hed-CS-NPs, and α-Hed-CS-CD147-NPs on SMMC-7721 cell line after treatment for 24 h (*n* = 3); and (**D**) cytotoxic effect of various formulations containing α-Hed on HSC cell line after incubation for 24 h (*n* = 3). *p < 0.05, **p < 0.01 versus α-Hed-CS-NPs.

*In vitro* cytotoxicity assays were performed using MTT with HepG2, SMMC-7721, and HSC cells treated with gradient concentrations of free α-Hed, α-Hed-CS-NPs, and α-Hed-CS-CD147-NPs for 24 h (Fig. 5). The *in vitro* cytotoxicity of the blank vehicles in HepG2 and SMMC-7721 cells were determined through the same treatment procedure using vacant CS-CD147-NPs. The cell viability in treatment with vacant CS-CD147-NPs was higher than 93% in HepG2 and SMMC-7721 cells incubated for 24 h, demonstrating that vacant CS-CD147-NPs were nontoxic. Free α-Hed, α-Hed-CS-NPs, and α-Hed-CS-CD147-NPs dose-dependently inhibited the *in vitro* proliferation of the three types of cells. The cytotoxicity of α-Hed-CS-CD147-NPs was higher than that of free α-Hed and α-Hed-CS-NPs on HepG2 cells. Similarly, SMMC-7721 cells treated with α-Hed-CS-CD147-NPs exhibited higher cytotoxicity than the cells with unmodified α-Hed-CS-NPs and free α-Hed. The increased cytotoxicity of CD147-modified NPs was correlated with antibody-mediated active targeting and high cellular uptake. HepG2 cells demonstrated higher sensitivity to α-Hed-CS-CD147-NPs than SMMC-7721 cells; thus, HepG2 cells were used to further investigate cell apoptosis, cellular uptake, and mechanisms.

HSC cells were used as control for CD147 non-expressing cell line to confirm the high cytotoxicity of CD147-mediated active targeting. After treatment with gradient concentrations of free α-Hed, α-Hed-CS-NPs, and α-Hed-CS-CD147-NPs, free α-Hed exhibited the lowest cytotoxicity. The antineoplastic activity of α-Hed-CS-CD147-NPs was similar to that of α-Hed-CS-NPs, illustrating that the strengthened anti-proliferation of α-Hed-CS-CD147-NPs was related to CD147-mediated active targeting for efficient cellular binding.

**Figure 6:**
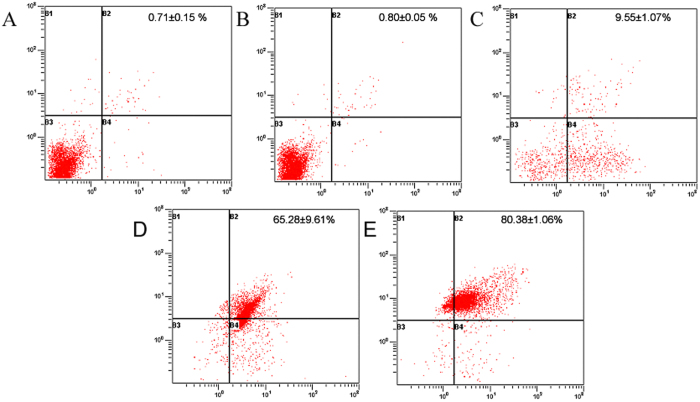
Apoptotic effects of different formulations on HepG2 cell line through Annexin V-PI method detected by flow cytometry after incubation for 24 h (*n* = 3). (**A**) Negative control; (**B**) void CS-CD147-NPs; (**C**) α-Hed; (**D**) α-Hed-CS-NPs; and (**E**) α-Hed-CS-CD147-NPs.

Programmed cell death, also called cell apoptosis, could be assessed using an Annexin V-PI kit. At the early stage of apoptosis, phosphatidylserine (PS) was oriented outward from the inner cell membranes and Annexin V exhibited high affinity for PS. Furthermore, PI traversed through the cell membrane of the apoptotic and dead cells, resulting in stained nucleus. Cells in different apoptotic stages could be distinguished by combining fluorescein FITC-labeled Annexin V and PI. In the scatter diagram of bivariate flow cytometry (Fig. 6), the lower left quadrant showed live cells, the upper right quadrant showed dead cells, also named as necrotic cells or late apoptotic cells, and the right down quadrant showed early apoptotic cells. After treatment with drugs, the cells were mostly distributed in the right upper and down quadrants, indicating that cell apoptosis induced drug-caused death of HepG2 cells. Nevertheless, the cells were not significantly different between treatment with crude NPs and negative control, attesting that vector used in this work demonstrated good biocompatibility without cytotoxicity. Cell apoptosis induced by NPs was higher than that induced by α-Hed, which may be associated with the passive targeting of NPs. Similarly, cell apoptosis induced by α-Hed-CS-CD147-NPs was more robust than that induced by α-Hed-CS-NPs, signifying the existence of antibody-mediated active targeting.

### Cellular uptake of NPs

**Figure 7:**
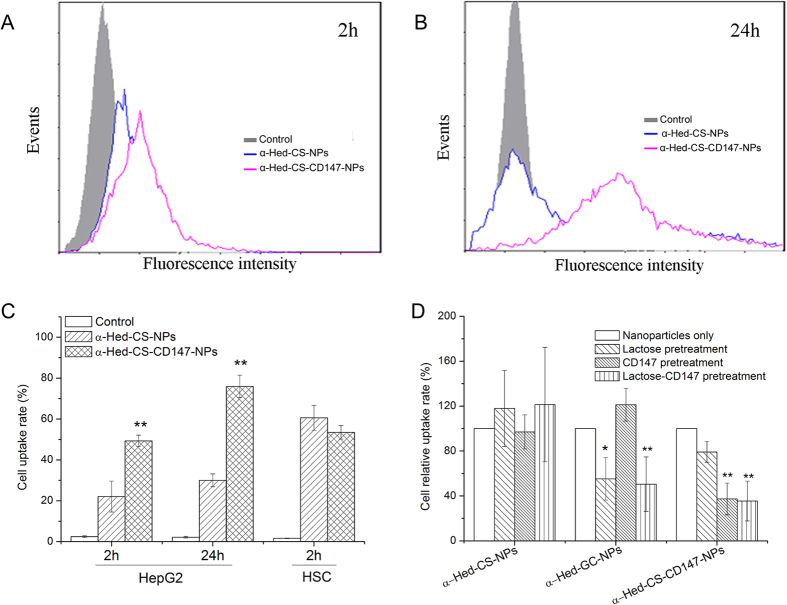
Cellular uptake of both nanoparticles in different cell lines or with various pretreatment as detected through flow cytometry. (**A**) Cellular uptake of unmodified and modified nanoparticles with FITC-labeled CD147 after incubation for 2 h; (**B**) HepG2 cell uptake of the two nanoparticles labeled with FITC after treatment for 4 h; (**C**) detailed HepG2 cell uptake rate of both nanoparticles for 2 and 24 h and the comparison of 2 h cell uptake between the over-expressed HepG2 cell line and the low-expressed HSC cell line; (**D**) competitive inhibitory effect of CD147 antibody on HepG2 cell uptake of α-Hed-CS-CD147-NPs with lactose on α-Hed-GC-NPs as positive control. *p < 0.05, **p < 0.01 versus control (nanoparticles only).

After 2 h of incubation (Fig. 7A), HepG2 cells exhibited a significantly higher uptake of α-Hed-CS-CD147-NPs than that of α-Hed-CS-NPs. After 24 h (Fig. 7B), the uptake of α-Hed-CS-CD147-NPs in HepG2 cells was higher than that of α-Hed-CS-NPs. By contrast, in HSC cells (Fig. 7C), no significant difference was observed between the two types of NPs. As CD147 exhibited no or low expression in HSC cells, the increased uptake of α-Hed-CS-CD147-NPs in HepG2 cells could be associated with the active targeting of the interaction between the antibody and the antigen.

As shown in Fig. 7D, the relative uptake of α-Hed-GC-NPs in HepG2 cells decreased because of lactose-induced competitive inhibition caused by the interaction between α-Hed-GC-NPs and the asialoglycoprotein receptor. This finding confirmed the feasibility of this experiment. Similarly, after pretreatment of cells with CD147 antibody, the cellular relative uptake of α-Hed-CS-NPs and α-Hed minimally changed but that of α-Hed-CS-CD147-NPs decreased, indicating CD147-mediated active targeting.

### HepG2 cellular binding and localization through confocal microscopy

**Figure 8:**
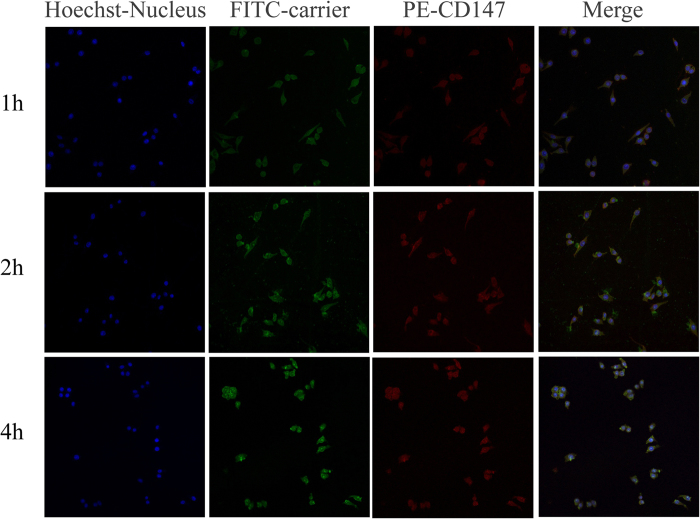
HepG2 cell uptake and binding after incubation with FITC-labeled α-Hed-CS-CD147-NPs for different periods through confocal microscopy. The blue, green, and red areas represent the nucleus, FITC-labeled α-Hed-CS-CD147-NPs, and PE-labeled CD147 antibody, respectively.

Figure 8 shows that the green fluorescence of α-Hed-CS-CD147-NPs accumulated around the cell membrane within 1 hour. Moreover, PE-labeled CD147 on the surface of α-Hed-CS-CD147-NPs accumulated around the cell membrane, which could be due to NP distribution for 1 h. After 2 h, the cytoplasm was oversupplied with large amounts of α-Hed-CS-CD147-NPs. As CD147 remained insulated around the cell membrane, it was present in the cell membrane rather than in the intercellular surroundings. After 4 h, most NPs were located in the nucleus and few PE-labeled CD147 was observed in the cytoplasm. This finding indicated that the specific recognition and interaction between antibody and antigen reached the saturation level, and CD147 was transferred into the cells with NPs.

### Effects of various endocytosis inhibitors

First, as endocytic uptake is energy dependent, the addition of the energy inhibitor sodium azide could significantly inhibit the process. Second, hyperosmotic conditions caused by high sucrose concentration hindered membrane internalization and the clathrin cycle of the coated pit pathway. This phenomenon reversed the internalization of low-density lipoproteins, randomly dispersed the low-density lipoprotein receptor on the membrane, and thus decreased the number of available coated pit for clathrin assembly. Third, chlorpromazine could effectively inhibit clathrin-mediated endocytosis, resulting in the accumulation of clathrin in the late-stage lysosome and inhibiting NP invagination of endocytosis. Fourth, methyl beta cyclodextrin could remove cholesterol molecules and disrupt the lipid raft structure of the cell membrane, leading to reduced membrane fluidity. Finally, wortmannin could effectively inhibit protein polarization involved in macropinocytosis.

**Figure 9:**
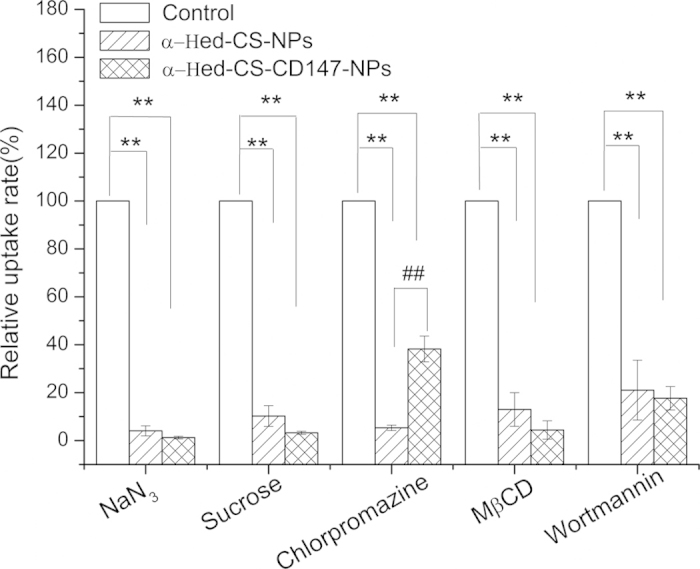
Influence of different endocytosis inhibitors on HepG2 cell uptake. HepG2 cells were pretreated with NaN_3_ (200 μg/mL), sucrose (0.45 M), chlorpromazine (7 μg/mL), methyl-β-cyclodextrin (MβCD, 3 nM), and wortmannin (500 nM) for 1 h and then incubated with FITC-labeled α-Hed-CS-NPs and α-Hed-CS-CD147-NPs for 2 h (*n* = 3). *p < 0.05 and **p < 0.01 versus control. ^#^p < 0.05 and ^#^^#^p < 0.01 versus α-Hed-CS-NPs.

After NaN_3_ pretreatment, the HepG2 cell uptake of α-Hed-CS-CD147-NPs decreased compared with that of α-Hed-CS-NPs, which could be due to the energy-dependent affinity of the antibody for the antigen (Fig. 9). In pretreatment with sucrose, the relative uptake rates were 10.23% ± 4.37% of α-Hed-CS-NPs and 3.23% ± 0.66% of α-Hed-CS-CD147-NPs but the difference was not significant. This finding revealed that these NPs entered into the cells through membrane internalization. In pretreatment with chlorpromazine, the relative uptake rates were 5.31% ± 1.14% of α-Hed-CS-NPs and 38.25% ± 5.37% of α-Hed-CS-CD147-NPs. This result indicated that both NPs required the clathrin-mediated pathway to enter into the cells. Moreover, clathrin may be beneficial to the interaction between CD147 antibody and antigen through clathrin accumulation feedback. With the destruction action of MβCD, the relative uptake rate of α-Hed-CS-CD147-NPs decreased compared with that of α-Hed-CS-NPs, indicating steric hindrance induced by CD147 modification. Furthermore, no significant difference was observed between the two NPs after wortmannin pretreatment. In general, endocytosis was relevant to giant phagocytosis.

The obtained results suggested that the endocytic uptake of drug-loaded NPs was energy dependent and required cell caveolae formation and clathrin-mediated pathway to pass into the cells. Hence, the stability and liquidity of the cell membrane must be further investigated. In the current work, the uptake mechanism of both NPs was partially similar, except for the influence of the clathrin-mediated pathway.

### Intracellular colocalization

**Figure 10:**
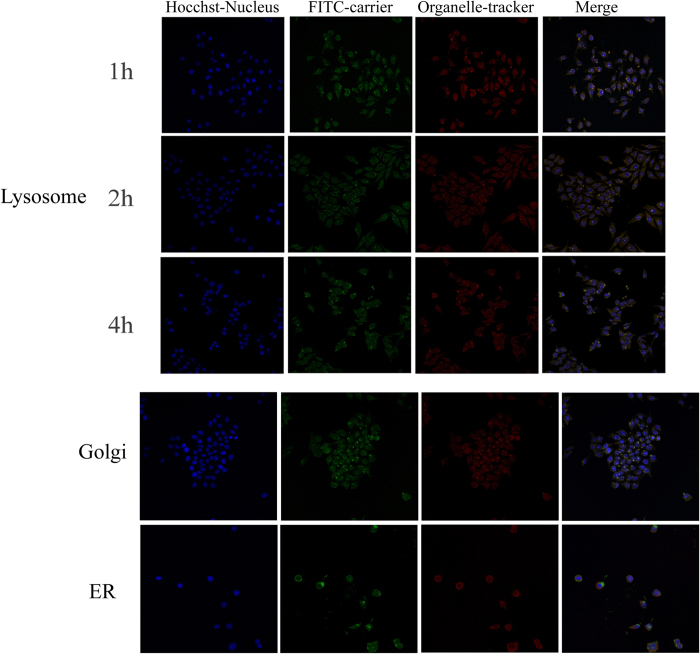
Sub-cellular localization of α-Hed-CS-CD147-NPs in HepG2 cells observed through confocal microscopy. The blue, green, and red areas represent the nucleus, FITC-labeled α-Hed-CS-CD147-NPs, and organelle-tracker (lysosome, Golgi, and endoplasmic reticulum) antibody, respectively. Lysosome was observed after incubation with α-Hed-CS-CD147-NPs for 1, 2, and 4 h. Golgi and endoplasmic reticulum were observed after incubation with α-Hed-CS-CD147-NPs for 2 h.

After HepG2 was incubated with α-Hed-CS-CD147-NPs for 1 h, NPs were mainly distributed around the cell membrane and few entered into the cells and captured by the lysosomes (Fig. 10). After 2 h, NPs were observed in the cytoplasm, lysosomes, acidic organelles, Golgi complex, and ER. Some parts of NPs were first swallowed into the lysosome for digestion, whereas some entered into the ER and transferred to the Golgi for transportation or secretion to the extracellular surroundings through endocytosis. After 4 h, part of the vectors entered into the nucleus but some NPs remained in the lysosomes, indicating lysosomal escape. The mechanism was probably similar to PEI “proton sponge effect.” After several protease actions, the release of acidic α-Hed led to decreased pH in the lysosome and the abundant free amino acids of the depolymerized CS captured numerous protons. This phenomenon resulted in chloride ion influx and thus promoted the osmotic pressure in the lysosomes. As a result, α-Hed and CS were released into the cytoplasm after the rupture of the lysosomes. As the Golgi and ER seemed to be involved in the drug delivery system in cells, caveolae-mediated endocytosis possibly existed.

### *In vivo* real-time imaging with NIR fluorescence

**Figure 11:**
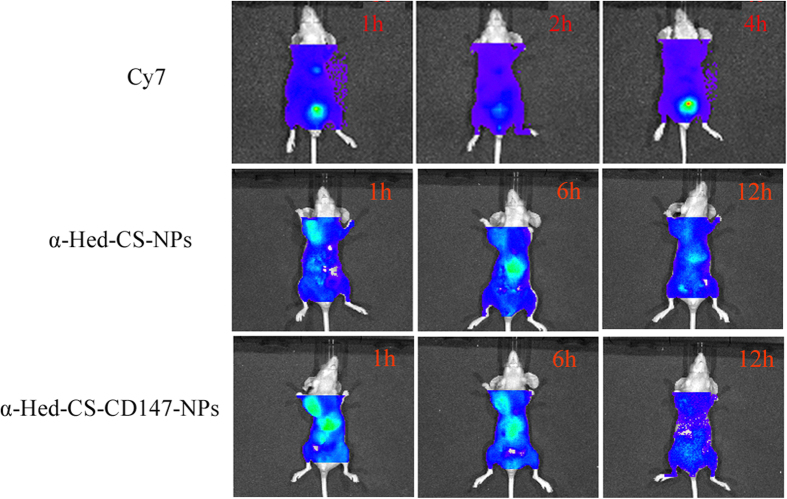
*In vivo* real-time imaging with NIR fluorescence of nude mice bearing HepG2 cells. Cy7 was given in the tail vein as negative control.

After 1 h of administration through the tail vein, both NPs were distributed to the whole body but the fluorescence of α-Hed-CS-CD147-NPs was more apparent than that of α-Hed-CS-NPs in tumor and liver cells, confirming superior active targeting. After 6 h, the fluorescence of both NPs weakened in cancer cells; the fluorescence of α-Hed-CS-NPs strengthened and that of α-Hed-CS-CD147-NPs minimally changed in the liver (Fig. 11). Most α-Hed-CS-CD147-NPs transferred and released into tumor cells though blood circulation and then released in tumor. With consistent administration, the drug concentration decreased in the liver. For vector-mediated inactive targeting, more α-Hed-CS-NPs accumulated in the liver than that in tumor cells. After 12 h, α-Hed-CS-NPs accumulated in the cancer cells, whereas α-Hed-CS-CD147-NPs started to abate until completely disappeared.

Lipophilic Cy7 was selected as the fluorescence marker and was jointly entrapped into CS with α-Hed. Free Cy7 was administered to nude mice as control to eliminate interference and distribute. For lack of sustained release, Cy7 underwent a rapid process of distribution, metabolism, and excretion. Accordingly, fluorescence distribution after 12 h was only observed within a short duration. After administering free Cy7 in the tail vein, fluorescence was detected in the abdomen after 1 and 4 h and then abated after 6 h. After 12 h, no fluorescence was observed in the liver or cancer cells.

## Conclusions

In this study, a monoclonal antibody was successfully modified to prepare CS NPs loaded with antitumor drugs. CD147-modified NPs achieved its full active targeting capacity to the liver tumor through antibody–antigen specific recognition. Antibody-modified void CS NPs exhibited no cytotoxicity to tumor cells, confirming its good biocompatibility. In addition, α-Hed-CS-CD147-NPs were captured into the cells through clathrin-mediated endocytosis and significantly affected stability and mobility. Antibody-modified NPs loaded with antitumor drugs could enhance further applications through antibody–antigen specific recognition.

## Additional Information

**How to cite this article**: Zhu, R. *et al.* CD147 monoclonal antibody mediated by chitosan nanoparticles loaded with a-hederin enhances antineoplastic activity and cellular uptake in liver cancer cells. *Sci. Rep.* **5**, 17904; doi: 10.1038/srep17904 (2015).

## Figures and Tables

**Figure 1:**
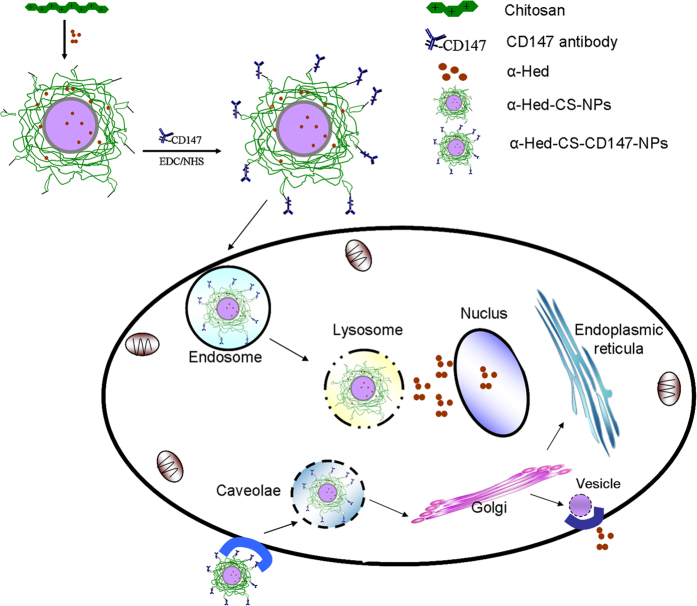
Schematic of cell uptake and intracellular distribution of α-Hed-CS-CD147-NPs.

## References

[b1] Vogelzang E. H. *et al.* Adalimumab trough concentrations in patients with rheumatoid arthritis and psoriatic arthritis treated with concomitant disease-modifying antirheumatic drugs. Ann Rheum Dis. 74, 474–475 (2015). 25433018 10.1136/annrheumdis-2014-206588

[b2] Buch M. H. *et al.* Updated consensus statement on the use of rituximab in patients with rheumatoid arthritis. Ann Rheum Dis. 70, 909–920 (2011). 21378402 10.1136/ard.2010.144998 PMC3086093

[b3] Strickler J. H. & Hurwitz H. I. Bevacizumab-based therapies in the First-Line Treatment of Metastatic Colorectal Cancer. Oncologist. 17, 513–524 (2012). 22477726 10.1634/theoncologist.2012-0003 PMC3336830

[b4] Martin-Castillo B. *et al.* Basal/HER2 breast carcinomas-Integrating molecular taxonomy with cancer stem cell dynamics to predict primary resistance to trastuzumab (Herceptin). Cell Cycle. 12, 225–245 (2013). 23255137 10.4161/cc.23274 PMC3575452

[b5] Yu M. *et al.* Determination of Saponins and Flavonoids in Ivy Leaf Extracts Using HPLC-DAD. J Chromatogr Sci. 53, 478–483 (2015). 24981979 10.1093/chromsci/bmu068

[b6] Rooney S. & Ryan M. F. Effects of alpha-hederin and thymoquinone, constituents of Nigella sativa, on human cancer cell lines. Anticancer Res. 25, 2199–2204 (2005). 16158964

[b7] Rooney S. & Ryan M. F. Modes of action of alpha-hederin and thymoquinone, active constituents of Nigella sativa, against HEp-2 cancer cells. Anticancer Res. 25, 4255–4259 (2005). 16309225

[b8] Cheng L. *et al.* The anticancer effect and mechanism of α-hederin on breast cancer cells. Int J Oncol. 45, 757–763 (2014). 24842044 10.3892/ijo.2014.2449

[b9] Mendel M. *et al.* Participation of extracellular calcium in α-hederin-induced contractions of rat isolated stomach strips. J Ethnopharmacol. 146, 423–426 (2013). 23274745 10.1016/j.jep.2012.12.023

[b10] Ganguly K. *et al.* Polysaccharide-based micro/nanohydrogels for delivering macromolecular therapeutics. J Control Release. 193, 162–173 (2014). 24845128 10.1016/j.jconrel.2014.05.014

[b11] Dash M., Chiellini F., Ottenbrite R. M. & Chiellini E. Chitosan-A versatile semi-synthetic polymer in biomedical applications. Progress in Polymer Science 26, 981–1014 (2011).

[b12] Agnihotri S. A., Mallikarjuna N. N. & Aminabhavi T. M. Recent advances on chitosan-based micro- and nanoparticles in drug delivery. J Control Release. 100, 5–28 (2004). 15491807 10.1016/j.jconrel.2004.08.010

[b13] Agnihotri S. A. *et al.* Recent advances on chitosan-based micro- and nanoparticles in drug delivery. J Control Release. 100, 5–28 (2004). 15491807 10.1016/j.jconrel.2004.08.010

[b14] Aminabhavi T. M. *et al.* Controlled release of therapeutics using interpenetrating polymeric networks. Expert Opin Drug Deliv. 12, 669–688 (2015). 25341410 10.1517/17425247.2014.974871

[b15] Aminabhavi T. M. *et al.* Guar gum as platform for the oral controlled release of therapeutics. Expert Opin Drug Deliv. 11, 753–766 (2014). 24650099 10.1517/17425247.2014.897326

[b16] Gupta S., Singh S. K. & Girotraa P. Targeting silymarin for improved hepatoprotective activity through chitosan nanoparticles. Int J Pharm Investig. 4, 156–163 (2014). 10.4103/2230-973X.143113 PMC4241620 25426436

[b17] Sun Y. *et al.* Newcastle disease virus vaccine encapsulated in biodegradable nanoparticles for mucosal delivery of a human vaccine. Hum Vaccin Immumother. 10, 2503–2506 (2014). 10.4161/hv.29201 PMC4896776 25424963

[b18] Aral C. & Akbuga J. Preparation and *in vitro* transfection efficiency of chitosan microspheres containing plasmid DNA:poly(L-lysine) complexes. J Pharm Pharm Sci. 6, 321–326 (2003). 14738712

[b19] Carrillo C. *et al.* Chitosan nanoparticles as non-viral gene delivery systems: determination of loading efficiency. Biomed Pharmacother. 68, 775–783 (2014). 25092239 10.1016/j.biopha.2014.07.009

[b20] Liu L. *et al.* TAT-LHRH conjugated low molecular weight chitosan as a gene carrier specific for hepatocellular carcinoma cells. Int J Nanomedicine. 10, 2879–2889 (2014). 24959076 10.2147/IJN.S61392 PMC4061174

[b21] Chaturvedi K. *et al.* Cyclodextrin-based siRNA delivery nanocarrier: a state-of-the-art review. Expert Opin Drug Deliv. 8, 1455–1468 (2011). 21867463 10.1517/17425247.2011.610790

[b22] Rudzinski W. E. *et al.* Chitosan as a carrier for targeted delivery of small interfering RNA. Int J Pharm. 399, 1–11 (2010). 20732398 10.1016/j.ijpharm.2010.08.022

[b23] Chaturvedi K. *et al.* Polymeric hydrogels for oral insulin delivery. J. Controlled Release. 165, 129–138 (2013). 10.1016/j.jconrel.2012.11.005 23159827

[b24] Hu Z. H. *et al.* Preparation of a novel liver-targeting nanoparticle of norcantharidin derivative and evaluation of its anti-tumour activity. J Exp Nanosc. 6, 183–199 (2011).

[b25] Wang Q. *et al.* Norcantharidin-associated galactosylated chitosan nanoparticles for hepatocyte-targeted delivery. Nanomedicine. 6, 371–381 (2010). 19699319 10.1016/j.nano.2009.07.006

[b26] Kim T. H. *et al.* Galactosylated chitosan/DNA nanoparticles prepared using water-soluble chitosan as a gene carrier. Biomaterials. 25, 3783–3792 (2004). 15020154 10.1016/j.biomaterials.2003.10.063

[b27] Park I. K. *et al.* Galactosylated chitosan-graft-dextran as hepatocyte-targeting DNA carrier. J Control Release. 69, 97–108 (2000). 11018549 10.1016/s0168-3659(00)00298-4

[b28] Park I. K. *et al.* Galactosylated chitosan-graft-poly (ethylene glycol) as hepatocyte-targeting DNA carrier. J Control Release. 76, 349–362 (2001). 11578748 10.1016/s0168-3659(01)00448-5

[b29] Gao S. Y. *et al.* Galactosylated low molecular weight chitosan as DNA carrier for hepatocyte-targeting. Int J Pharm. 255, 57–68 (2003). 12672602 10.1016/s0378-5173(03)00082-6

[b30] Zhu H. Y. *et al.* Folate-modified chitosan micelles with enhanced tumor targeting evaluated by near infrared imaging system. Carbohydrate Polymers. 86, 1118–1129 (2011).

[b31] Guan M. *et al.* N-Trimethyl Chitosan Nanoparticle–encapsulated Lactosyl-Norcantharidin for Liver Cancer Therapy with High Targeting Efficacy. Nanomedicine. 8, 1172–1181 (2012). 22321383 10.1016/j.nano.2012.01.009

[b32] El-Shabouri M. H. *et al.* Positively charged nanoparticles for improving the oral bioavailability of cyclosporin-A. Int J Pharm. 249, 101–108 (2002). 12433438 10.1016/s0378-5173(02)00461-1

[b33] Zhang X. *et al.* Autophagy induced by baicalin involves downregulation of CD147 in SMMC-7721 cells *in vitro*. Oncol Rep. 27, 1128–1134 (2012). 22200845 10.3892/or.2011.1599 PMC3583557

[b34] Arya G., Vandana M., Acharya S. & Sahoo S. K. Enhanced antiproliferative activity of Herceptin (HER2)-conjugated gemcitabine-loaded chitosan nanoparticle in pancreatic cancer therapy. Nanomedicine. 7, 859–870 (2011). 21550422 10.1016/j.nano.2011.03.009

[b35] Niu H. *et al.* Treatment of (131)I-labeled anti-CD147 monoclonal antibody in VX2 carcinoma-induced liver tumors. Oncol Rep. 30, 246–252 (2013). 23612800 10.3892/or.2013.2418

[b36] Zhu Q. L. *et al.* Low-density lipoprotein-coupled N-succinyl chitosan nanoparticles co-delivering siRNA and doxorubicin for hepatocyte-targeted therapy. Biomaterials. 35, 5965–5976 (2014). 24768047 10.1016/j.biomaterials.2014.03.088

[b37] Zhu R. *et al.* Preparation and *in vitro* study of α-Hederin -Loaded Chitosan nanoparticles. Chinese Traditional Patent Medicine. 35, 1906–1911 (2013).

[b38] Zhang Y. T. *et al.* Evaluation of transdermal salidroside delivery using niosomes via *in vitro* cellular uptake. Int J Pharm. 478, 138–146 (2014). 25448576 10.1016/j.ijpharm.2014.11.018

[b39] Warburton M. J. & Wynn C. H. The hyperactivity of hamster fibroblast lysosomal enzymes after endocytosis of sucrose. Biochem Biophys Res Commun. 70, 94–100 (1976). 1275945 10.1016/0006-291x(76)91113-x

[b40] Ohara K., Kohno M., Hamada T. & Kawakami K. Entry of a cationic lytic-type peptide into the cytoplasm via endocytosis-dependent and -independent pathways in human glioma U251 cells. Peptides. 50, 28–35 (2013). 24095870 10.1016/j.peptides.2013.09.015

[b41] Plazzo A. P. *et al.* Uptake of a fluorescent methyl-β-cyclodextrin via clathrin-dependent endocytosis. Chem Phys Lipids. 16, 505–511 (2012). 22503802 10.1016/j.chemphyslip.2012.03.007

[b42] Chiu Y. L. *et al.* The characteristics, cellular uptake and intracellular trafficking of nanoparticles made of hydrophobically-modified chitosan. J Control Release. 146, 152–159 (2010). 20580915 10.1016/j.jconrel.2010.05.023

